# Personality differences in early versus late suicide attempters

**DOI:** 10.1186/s12888-016-0991-6

**Published:** 2016-08-09

**Authors:** Ute Lewitzka, Sebastian Denzin, Cathrin Sauer, Michael Bauer, Burkhard Jabs

**Affiliations:** 1 Department of Psychiatry and Psychotherapy, University Hospital Carl Gustav Carus, Technische Universität Dresden, Fetscherstr. 74, D-01307 Dresden, Germany; 2 Technische Universität Dresden, Dresden, Germany; 3 Psychiatric Department of the Municipal Hospital Dresden-Neustadt, Dresden, Germany

**Keywords:** Personality, Suicide attempt, Temperament and character Inventory, Impulsivity

## Abstract

**Background:**

Suicidality is an individual behaviour caused by a complex framework of internal and external factors. The predictive values of personality traits for a suicide attempt have been demonstrated, especially in conjunction with Cloninger’s TCI and impulsivity. Two issues remain unsolved, namely whether these traits alter over time after a suicide attempt, and how they may be influenced by depressive symptoms.

**Methods:**

We studied two patient cohorts: one sample of 81 patients after a suicide attempt no longer than 3 months previously (SA early) and another sample of 32 patients whose attempt had taken place more than 6 months previously (SA late). We carried out structured interviews with these subjects addressing diagnosis (MINI), suicidality (Scale for suicide ideation), depression (HAMD-17), temperament and character inventory (TCI), and impulsivity (BIS-10). Data analysis was done using SPSS 16.0.

**Results:**

Our two groups did not differ significantly in sociodemographics or suicidality. However, patients in the SA early group were significantly more depressed (*p* < 0.001), and scored lower in reward dependence (*p* < *0.001*) and persistence (*p* = *0.005*) but higher in harm avoidance (*p* < *0.001*); they did not differ significantly in impulsivity (*p* < *0.01*). Reward dependence, persistence, and harm avoidance remained significantly different between the two groups after controlling for depressive symptoms.

**Conclusions:**

Our findings suggest that some personality traits vary after a suicide attempt. Further investigations are necessary to verify our results, ideally in longitudinal studies with larger, carefully-described cohorts. It would be also clinically important to investigate the influence of therapeutic strategies on the variability of personality traits and their impact on suicidal behavior.

## Background

Suicidality is a complex phenomenon associated with substantial individual and societal burden [1]. Suicide is one of the top 20 leading causes of death globally for all ages. Nearly a million people die every year from suicide [2]. There are estimates that 58,000 people kill themselves within the European Union and about 10.000 people die by suicide in Germany [3]. What is of most worrying is the high proportion of suicides in younger age groups in relation to other causes of death. For example, among individuals aged 15 to 39 years, suicide is the second leading cause of death after accidents [4]. However, the current number of suicide attempts in this age range remains unclear. It is estimated that suicide attempts occur 10 to 20 times more often than suicides [5]. Suicidality is caused by multiple factors, one of the most common being psychiatric disease [6]. Epidemiological research reveals that approximately 90 % of individuals who kill themselves suffered from a psychiatric disorder at the time of death [7].

Over the past decade, several working groups have focused on psychopathological and neurobiological risk factors for suicide. A suicide attempt in the past, male sex, older age, comorbidity, and anomalies in the serotoninergic system are well-known and established predictors for suicides, especially in patients with mood disorders [6, 8–12]. Factors considered risk factors for suicide attempts are psychiatric diseases, age below 25 years, female sex, severe physical disease, suicide attempts in the past, stressful life events, loneliness, lower socio-economic income, and different stages of individual development [13–16].

Personality and temperamental traits have also been a focus of research, as they may facilitate the early identification of suicidal risk. Studies on personality and suicidality have examined traits that increase the risk of attempted suicide such as anger, impulsivity, aggression, and anxiety [17] and especially the two factors harm avoidance and novelty seeking [18] of the Temperament and Character Inventory (TCI, [19]). Among younger individuals, impulsive-aggressive traits have been shown to be especially closely associated with a higher risk of suicide [20]. The study of personality traits is challenging, as there are many different measurements used involving various methodological limitations, and the findings regarding the stability of personality have been inconsistent.

There is also wide variability in the measurement tools used to assess personality traits, temperament, and suicidality.

Another important factor is the stability of personality markers and temperament on the one hand, and their influence on suicidality on the other.

A study by McCrae and Costa [21] demonstrated that personality traits appear to be stable around age 30. Additionally, in their meta-analysis of 152 prospective studies, Roberts and Delvecchio [22] demonstrated that beyond age 50, personality traits are stable. Other studies have shown that the long-term consistency of personality traits diminishes as we age [23]. Determining the stability of personality traits such as impulsivity would be particularly key to improving the clinical assessment of the risk of suicide attempts.

To the best of our knowledge, no study before now has addressed the long-time-stability of TCI factors. The longest interval of a re-test in a representative group (normal population) was 12 months [24]; it exhibited highly stable factors. Studies in different languages have shown a range of moderate reliability coefficients 0.45 to 0.93 [25–32]. According to studies by Richter et al. [25], Borman et al. [33] and Boz et al. [34], we assume that factors like self-directedness or harm-avoidance may influence the variability of TCI in people with mental disorders. It is not well known whether this variability is also observed in healthy controls and whether it is caused by therapeutic strategies or the illness itself.

There are few studies to date that have investigated the association between changes in impulsivity and the time of a suicide attempt. Corruble et al. [35, 36] investigated 36 suicide attempters who were depressed and detected a significant drop in overall impulsivity as measured by the Barratt Impulsivity Scale (BIS, [37]), as well as a decrease in 3 subscales 4 weeks after the suicide attempt. Similarly, another study of 39 suicide attempters [38] found lower impulsivity scores immediately after the attempt and 13.5 days later. In contrast, Jallade et al. [39] reported stable impulsivity scores (as measured by the BIS) in a group of 26 suicide attempters on the day of the suicide attempt compared to a week later.

Another variable to consider is how depressive symptoms lead to changes in personality traits. Several studies have similarly examined the association between depressive symptoms and personality traits measured by the TCI that yielded convergent results. That is, personality traits as measured by the TCI were stable even during a change in depressiveness; only the specific subscale harm avoidance was significantly influenced by depressive symptoms [40–44]. The findings from studies investigating specifically an association between impulsivity and depressive symptoms are more heterogeneous, with some studies demonstrating a negative association between impulsivity and depression (e.g., [45, 46]) while others describe a positive association (e.g., [36, 47, 48]).

The present study aimed to investigate the extent to which the time interval after a suicide attempt influences personality traits in patients who attempt suicide. We expected to find that the personality traits of patients whose suicide attempt was recent will differ from those whose attempt took place more than 6 months prior and that differences in personality traits are independent from the severity of depressive symptoms.

## Methods

### Participants and recruitment

Due to our design, we compared two different patient groups but not applying pre- versus post-suicide attempt measures on an individual basis. Patients with a recent suicide attempt occurring less than 3 months before their participation in this study (SA early) were recruited from five centers (Luebeck, Dresden, Berlin, Bonn, Nuremburg) participating in “The German Research Network on Depression” [49]. Patients recruited from these centres had participated in a randomised double-blind placebo controlled-trial conducted to investigate the anti-suicidal effect of lithium in patients with affective disorders (a lithium-intervention study). That study’s methods and results are described elsewhere [50, 51]. Our second patient group consisted of patients whose suicide attempt had occurred more than 6 months before their participation in this study (SA late); they were recruited from the outpatients department in Dresden, Germany. Both patient groups were recruited during the same time period. A complete description of the study was provided to participants and written informed consent was obtained. The study protocol was approved by the local research ethic boards at all participating centres.

### Inclusion and exclusion criteria

Table 1 provides a detailed overview of our inclusion and exclusion criteria. Briefly, patients were included in the study if they met DSM-IV criteria for an affective spectrum disorder and had attempted suicide within the past 3 months, or before the 6 months prior to participating in this study (Table 1).

**Table 1:** Inclusion/Exclusion criteria

	SA early	SA late
Inclusion criteria	• Suicide attempt < 3 months • Age >18 • Affective spectrum disorder (major depression, dysthymia, depression NOS, adjustment disorder with depression • Ability to understand and provide informed consent	• Suicide attempt > 6 months; < 5 years • Age >18 • Ability to understand and provide informed consent
Exclusion criteria	• Indication for long-term lithium therapy or contraindications for lithium therapy • Lithium therapy • Organic brain diseases, schizophrenia, unstable physical illness, • Kidney abnormalities, Mb. Addison, diseases with electrolyte disturbances, thyroid diseases, pregnancy and lactation	• Organic brain diseases, schizophrenia, instable physical illness • Lithium therapy

*Abbreviations*: *SA* suicide attempt

### Clinical and personality assessment

Detailed information of the patient’s psychiatric history and sociodemographics were obtained by trained psychiatrists using the patient’s interview material from previous studies (SA early, SA late) as well as all additional information from their charts (SA late). Clinical information was obtained using the Mini-International Neuropsychiatric Interview [52], the Hamilton Rating Scale for Depression [53], and the Scale for Suicide Ideation [54]. The Temperament and Character Inventory [55] and Barratt Impulsivity Scale [56] were used to assess personality and temperamental traits (Table 2).

**Table 2:** Overview clinical and behavioural assessment

Scale	Reference	Measure	Items	Remarks
MINI Mini-International Neuropsychiatric Interview	Sheehan et al., 1998	15 major adult Axis I diagnostic categories, 1 Axis II disorder and suicidality according ICD 10 and DSM IV		Semi-structured interview
HAM-D21 Hamilton Rating Scale for Depression	Hamilton 1969	Depressive symptoms	21 items, 5 point scale rating from 0 to 4 overall score: >23: very severe; 19–22: severe, 14–18: moderate, 8–13: mild, 0–7: no depression	Multiple choice questionnaire expert rating
SSI Scale for Suicide Ideation	Beck et al., 1979	Suicidal ideation; 3 dimensions: active suicidal desire, specific plans for suicide, passive suicidal desire	19-items; 3 point scale rating from 0 to 2; range of score: 0 –38, higher scores indicate greater suicidal ideation	Semi-structured interview
TCI Temperament and Character Inventory	Cloninger et al. 1994	7 dimensions: temperament (Novelty seeking, Harm Avoidance, Reward Dependence, Persistence) and character traits (Self-directedness, Cooperativeness, Self-transcendence)	Each of the dimension has a varying number of subscales, e.g.,: Novelty seeking (NS): Exploratory excitability (NS1), Impulsiveness (NS2), Extravagance (NS3), Disorderliness (NS4),	Self- questionnaire in total 240 yes/no items
BIS Barratt Impulsivity Scale	Barratt 1965	Impulsivity; 3 dimensions: non-planning, motor impulsiveness, attentional impulsiveness	30-items; 4 point rating from 1 to 4 Overall score reflects the intensity of impulsiveness	Self-rating scale evaluation of lasting personality-markers

All diagnostic and treatment information were reviewed by a panel of experienced psychiatrists within a consensus procedure. To ensure a high level of inter-rater reliability, periodical training sessions with all involved psychiatrists were conducted.

### Statistics

Analyses were conducted using SPSS 16.0 for Windows (SPSS Inc., Chicago IL, USA). Group differences were evaluated using chi-square (in case of categorical variables) and Mann–Whitney *U*-test (in case of continuous variables) for independent samples. The level of significance was set to *p* = .*05* (two-sided). Multivariate analysis of covariance was applied to examine group differences in TCI and BIS scales and the influence of depression (HAM-D) on personality traits by including the HAM-D total score as covariate.

Associations between psychopathological symptoms, behavioral and trait variables with suicidality variables were examined via partial correlation analysis.

## Results

### Sociodemographic factors

The present analysis included 81 participants from the SA early group and 32 from the SA late group (Tables 3 and 4). Sociodemographics and affective diagnoses did not differ significantly between early and late SA groups (please see Tables 3 and 4). The time interval between the interview and suicide attempt was on average (median) 26 days (SA early) versus 700 days (SA late).

**Table 3:** Clinical variables

Diagnoses N (%)	SA early (*N* = 81)	SA late (*N* = 32)	*p*	
Major depression	58 (71.6)	19 (59.4)	0.37	Chi^2^ = 5.402 df = 4 effect size Cohen’s w = 0.21
Dysthymia	5 (6.2)	0		
Adjustment Disorder	18 (22.2)	8 (25)		
Personality disorder	0	1 (3,1)		
No psychiatric diagnoses	0	4 (12.5)		

*Abbreviations*: *SA* suicide attempt

*df* degrees of freedom; *p* = .05 (two-sided) in Chi-square

**Table 4:** Sociodemographic variables

Sociodemographic variables	SA early (*N* = 81)	SA late (*N* = 32)	*p*	
Age mean ± SD	39.4 ± 13.5	41.9 ± 13.9	0.5	U = 1205.0; Z = −0.675 effect size *r* = 0.06 (*r* = Z/√N)
Sex (female/male) N (%)	51/31 (62.3/37.8)	20/12 (62.5/37.5)	0.98	Chi^2^ = 0.001; effect size w = 0 df = 1
Marital status N (%):			0.53	Chi^2^ = 2.23 effect size w = 0.15 df = 3
Single	31 (37.8)	11 (34.4)		
Married	26 (31.7)	7 (21.9)		
Divorced	21 (25.6)	11 (34.4)		
Widowed	4 (4.9)	3 (9.4)		
Highest qualification N (%):			0.13	Chi^2^ = 4.101 effect size w = 0.25 df = 2
Secondary general school	28 (34.1)	5 (15.6)		
Intermediate secondary school	35 (42.7)	16 (50)		
Grammar school	19 (23.2)	11 (34.4)		
Occupational status N (%):			0.11	Chi^2^ = 10.52 effect size w = 0.31 df = 6
Employed				
Full-time	36 (43.9)	13 (40.6)		
Part-time	9 (11.0)	1 (3.1)		
Unemployed	13 (15.9)	9 (28.1)		
Homemaker	3 (3.7)	0		
In apprenticeship	15 (18.3)	3 (9.4)		
Invalidity pension	3 (3.7)	1 (3.1)		
Retirement	3 (3.7)	5 (15.6)		
Living situation N (%):			0.37	Chi^2^ = 3.2 effect size w = 0.19 df = 3
Alone	33 (40.2)	14 (43.8)		
With both parents	9 (11.0)	1 (3.1)		
With husband/wife/common-law	37 (45.1)	17 (53.1)		
With other person	3 (3.7)	0		

*Abbreviations*: *SA* suicide attempt

*df* degrees of freedom; *p* = .05 (two-sided) in Chi-square

### Clinical features

Suicidal ideation was low and was not significantly different between early and late SA groups (*p* = 0.66). Specifically, patients from the SA early group revealed mean suicidality values of 5.95 (±7.69), whereas patients from the SA late group had mean values of 4.78 (±6.38), reflecting a low level of suicidal ideation in both groups.

Depressive symptoms (HAMD overall score) differed significantly between groups. Specifically, patients from the SA early group had mean values of 14.1 (±6.95); those from the SA late group had mean values of 7.25 (±4.91, *p* = <*0.001*), respectively. Therefore, the HAMD total score was included as a covariate in the analysis of covariance.

### Personality traits measured with TCI

Patients with a recent suicide attempt (SA early) were more pessimistic and more fearful (harm avoidance), more withdrawn, and more independent (reward dependence), as well as more inactive and more aimless (persistence) than patients in the SA late group. The two groups were most significantly differentiated by the dimensions of harm avoidance and reward dependence (*p* < *0.001*) (Table 5).

**Table 5:** Multivariate analysis of variance of TCI scales

Dependent variable	SA early (mean ± SD)	SA late (mean ± SD)	F(2,111)	*p*	Partielles Eta-Quadrat	Cohen’s d
Novelty seeking	19.26 ± 4,3	19.84 ± 5,4	0.19	0,830	0.003	0.125
Harm avoidance	18.94 ± 3,2	12.55 ± 4,3	22.13	<0,001	0.285	1.806
Reward dependence	12.5 ± 2,5	14.81 ± 3,5	7.71	0,001	0.122	0.821
Persistence	3.38 ± 1,4	4.41 ± 1,8	5.37	0,006	0.088	0.677
Self-directedness	22.43 ± 5,2	24.38 ± 4,2	1.75	0,179	0.031	0.394
Cooperativeness	22.36 ± 3,3	21.22 ± 3,6	1.74	0,181	0.030	0.337
Self-transcendence	11.15 ± 5	11.16 ± 4,3	0.05	0,954	0.001	0.002
Subscale impulsivity	5.92 ± 1.91	4.21 ± 2.85	7.35	0,001	0.117	0.774

*Abbreviations*: *SA* suicide attempt, *SD* standard deviation

p: significance of corrected model with groups as factor and HAMD17 as covariate

group as factor: F(7, 105) = 9,427; Pillai’s trace = 0,386; Wilk’s lambda = 0,614; *p* < 0,001

HAMD17 as covariate: F(7, 105) = 1,120; Pillai’s trace = 0,069; Wilk’s lambda = 0,936; *p* = 0,356

d.f. degrees of freedom = 2

### Impulsivity

Patients in the SA early group were in the mean more impulsive in terms of attentional impulsiveness as well as their overall BIS scores than the SA late group patients (please see Table 6). The SA early group scored significantly higher than the SA late group in only the TCI subscale for impulsivity (NS2) (please see Table 5). The differences in BIS overall scores and BIS subscales were not significant (Table 6).

**Table 6:** Multivariate analysis of variance of BIS scales

Dependent variable	SA early (mean ± SD)	SA late (mean ± SD)
BIS “Non-Planning”	24.02 ± 4.52	22.97 ± 3.51
BIS “Motor Impulsiveness”	23.46 ± 5.13	21.38 ± 4.61
BIS “Attentional Impulsiveness”	28.13 ± 4.89	25.91 ± 3.56
BIS Overall Score	75.62 ± 11.3	70.25 ± 9.25

*Abbreviations*: *SA* suicide attempt, *SD* standard deviation

group as factor: F(4, 108) = 0.766; Pillai’s trace = 0.028; Wilk’s lambda = 0.972; *p* = 0.550

HAMD17 as covariate: F(4, 108) = 1.186; Pillai’s trace = 0.042; Wilk’s lambda = 0.958; *p* = 0.321

We detected a low correlation between the TCI subscale for impulsivity (NS2) and BIS overall score (Pearson coefficient *r* = 0.232; *p* = *0.013*; *n* = 114) (Table 7).

**Table 7:** Correlations of TCI scales and BIS to HAM-D17 (*N* = 114)

Scale	Pearson’s correlation coefficient	*p* for two-sided significance
Novelty seeking	0.073	0.514
Harm avoidance	0.451	0.023
Reward dependence	0.058	0.607
Persistence	0.108	0.333
Self-directedness	0.156	0.16
Cooperativeness	0.149	0.183
Self-transcendence	−0.006	0.956
TCI subscale (impulsivity)	0.057	0.546
BIS overall score	0.14	0.136
BIS “Non-Planning”	0.003	0.975
BIS “Motor Impulsiveness”	0.144	0.127
BIS “Attentional Impulsiveness”	0.173	0.066

## Discussion

This study examined sociodemographic, clinical, and personality factors among patients with affective spectrum disorders who had suffered a recent or past suicide attempt. Both patient groups were similar in sociodemographic and clinical variables. Despite the difference in the timing after the suicide attempt, the groups did not differ significantly in suicidal ideation.

### TCI

Patients whose suicide attempt occurred 6 and more months and no longer than 5 years from their time of interview demonstrated significant differences in personality traits from patients whose suicide attempt was more recent (<3 months). There is strong evidence that higher levels of harm avoidance are associated with a higher risk for suicide attempts (e.g., [14, 57–61]). Specifically: in our study, our SA late group patients were lower in harm avoidance and higher in reward dependence and persistence, thus supporting our hypothesis. According to Cloninger’s theory, people high in reward dependence are warm, sentimental, pleasant, sociable, sensitive, and socially dependent. People high in persistence are eager, hard workers, ambitious, and more perfectionists. Harm avoidance includes four special aspects (subscales) described as anticipatory worry (HA 1), fear of uncertainty (HA 2), shyness with strangers (HA 3) and fatigability and asthenia (HA 4). Our findings suggest that patients whose suicide attempt was more recent are more pessimistic, fearful (harm avoidance), withdrawn (reward dependence), less demanding and more inactive (persistence) than patients whose suicide attempt was less recent.

Lower levels of harm avoidance and higher levels of reward dependence and persistence may be protective, but further research is needed to confirm this, as these assessments were conducted after a suicide attempt. The variability identified in the reward dependence and persistence dimensions are in line with findings from Richter et al. [25], who also described these dimensions as being moderately stable.

### TCI and suicide attempts

There is strong evidence that a higher level of harm avoidance is associated with a higher risk for suicide attempts (see above). However, many of those studies are limited by small patient cohorts and cross-sectional designs (e.g., [58, 59]).

Our investigation shows that patients whose suicide attempt was recent are more pessimistic, more fearful (harm avoidance), more withdrawn and independent (reward dependence) as well as more inactive and aimless (persistence) than patients who attempted suicide a longer time ago. These three dimensions seem to be state-dependent, which supports several aethiopathogenesis models (e.g., crisis model, latent trait model) of suicidal behavior (e.g., [62, 63]).

### Impulsivity

Patients whose suicide attempt was recent were not significantly higher in BIS impulsivity, but they were higher in TCI-impulsivity (NS2) than patients whose suicide attempt was further in the past. A low correlation between the BIS and TCI subscales may refer to low validity of TCI subcales [25]. There is more convincing evidence of the BIS’ sufficient validity [37]. One may assume that results based on BIS are more meaningful. Our results suggest that impulsivity is stable as a trait [64], a finding that stands in contrast to the few studies revealing lower impulsivity in conjunction with time after suicide attempt [35, 36, 65].

### Depression

Patients whose suicide attempt was recent presented significantly higher levels of depressive symptoms, an observation consistent with many other reports indicating that most patients remain depressed following a suicide attempt. The patient’s mood usually improves after a therapeutic intervention [38, 66].

In this analysis, depressive symptoms displayed no influence on the relationship between personality traits and the interval from suicide attempt. Harm avoidance was significantly associated with depressive symptoms, consistent with previous reports and expected, as many behaviours of harm avoidance overlap with symptoms of depression [40, 44].

On the other hand, longitudinal studies investigating TCI variability show relatively high stability for the dimension harm avoidance [24, 33, 34] inconsistent with our findings. However, those studies did not take suicidality or depressive symptoms into account.

Our findings suggest that depression was not the only parameter that varied in the cohort further from their suicidal event [67]. Javdani et al. [68] recently demonstrated that several personality traits (including impulsivity) were stronger predictors of suicide attempts than depressive symptoms. Their study also demonstrated that depressive symptoms only predict general suicide risk (i.e., ideation and plans), but not suicidal behaviors. This is an important finding for clinicians when identifying and evaluating suicide risk. In addition to depressive symptoms, personality factors should also be monitored.

As some of these personality traits appear to vary over time and may indicate a higher suicide risk, it is worthwhile investigating potential interventional strategies incorporating these traits. For example, it may be useful to emphasize specific protective traits such as cognitive impulsivity, harm avoidance, persistence, and reward dependency.

## Strengths and limitations

The main limitation of the present study is its cross-sectional design, which makes some of our interpretations difficult. Our study design did not allow us to identify *when* and which personality factors changed over time (such as harm avoidance and impulsivity, reward dependence, and persistence). In addition, we did not include a healthy control group. Some patient-cohort differences may have influenced our findings. For example, the SA late group patients were only recruited from one centre in Dresden, whereas patients from the other group were recruited from 5 different centres. Moreover, we had no data on psychiatric comorbidities or lifetime suicidality, or substance abuse available for comparison. However, these groups were sociodemographically similar. The SA early group contained both inpatients and outpatients, and many of them were on medication and/or undergoing psychotherapy, factors that could have influenced the clinical variables under investigation.

Future research should identify when specific traits change and investigate other influencing factors.

Positive aspects of our study are that we employed a wide range of standardised, valid, and reliable measurement tools and had diagnoses confirmed by psychiatrists rather than only trained raters.

## Conclusions

Suicidality is caused by a complex framework of internal and external factors. In this study we have identified specific personality traits that could help to deepen our knowledge about the development of suicidality. Our findings suggest that two groups whose interval to their suicide attempt varies also differ in some personality traits. If future studies suggest that several personality traits are changeable over time, these factors could be useful tools when assessing suicidal risk and considering therapeutic strategies.

In the light of these findings we recommend that clinicians pay close attention to specific personality traits such as pessimism, fearfulness, withdrawn behaviour, and fatigability (harm avoidance), but also to aspects of persistence and reward dependence when conducting assessments on suicidal patients.

Although assessment tools are helpful, we would like to emphasise the clinical importance of a reliable and trustworthy relationship with the patient experiencing a suicidal crisis. Future studies should also investigate a larger group of patients longitudinally. It would also be clinically relevant to investigate the influence of therapeutic strategies on the variability of personality traits and their impact on suicidal behavior.
